# Setting Research Priorities for Preconception Care in Low- and Middle-Income Countries: Aiming to Reduce Maternal and Child Mortality and Morbidity

**DOI:** 10.1371/journal.pmed.1001508

**Published:** 2013-09-03

**Authors:** Sohni Dean, Igor Rudan, Fernando Althabe, Aimee Webb Girard, Christopher Howson, Ana Langer, Joy Lawn, Mary-Elizabeth Reeve, Katherine C. Teela, Mireille Toledano, Chandra-Mouli Venkatraman, José M. Belizan, Josip Car, Kit Yee Chan, Subidita Chatterjee, Stanley Chitekwe, Tanya Doherty, France Donnay, Majid Ezzati, Khadija Humayun, Brian Jack, Zohra S. Lassi, Reynaldo Martorell, Ysbrand Poortman, Zulfiqar A. Bhutta

**Affiliations:** 1 Division of Women & Child Health, the Aga Khan University, Karachi, Pakistan; 2 Centre for Population Health Sciences, The University of Edinburgh Medical School, Edinburgh, United Kingdom; 3 Institute for Clinical Effectiveness and Health Policy, Buenos Aires, Argentina; 4 Hubert Department of Global Health, Rollins School of Public Health, Emory University, Atlanta, United States of America; 5 Global Programs, March of Dimes Foundation, White Plains, United States of America; 6 Department of Global Health and Population, Harvard School of Public Health, Harvard University, Boston, United States of America; 7 Saving Newborn Lives- Save The Children, Cape Town, South Africa; 8 Global Health Program, Bill and Melinda Gates Foundation, Seattle, United States of America; 9 MRC-HPA Centre for Environment and Health, Department of Epidemiology and Biostatistics, School of Public Health, Imperial College London, United Kingdom; 10 Department of Reproductive Health and Research, World Health Organization, Geneva, Switzerland; 11 Global eHealth Unit, Imperial College, London, United Kingdom; 12 Nossal Institute of Global Health, University of Melbourne, Melbourne, Australia; 13 Virtual Institute for Advancement of Women, Children and Young People, India and Thailand; 14 United Nations Children's Fund, Malawi; 15 Health Systems Research Unit, Medical Research Council, South Africa and School of Public Health, University of the Western Cape, South Africa; 16 Department of Family Medicine, Boston University, Boston, United States of America; 17 International Genetic Alliance of parent and patient organizations and Preparing for Life, the Netherlands

## Abstract

Sohni Dean and colleagues report their CHNRI exercise that developed health research priorities for effective pre-conception care in low- and middle-income countries.

*Please see later in the article for the Editors' Summary*

**Table:** Box 1. Preconception Risks and Interventions

Risks that could be reduced/mitigated	Any known interventions?
**BIOMEDICAL**	
**Maternal pre-pregnancy overweight (body mass index)**	Weight loss programs for adults and post-partum women incorporating diet and/or exercise
**Poor nutrition and pre-pregnancy underweight**	**Micronutrient supplementation**
Chronic medical conditions (diabetes, hypertension, etc.)	**Counseling and optimizing glycemic control for pre-pregnancy diabetes**
Infectious diseases especially sexually transmitted infections	Immunization, screening and treatment
Genetic disorders, consanguinity	Genetic counseling and screening
Mental health disorders	
Advanced maternal age	
**SOCIAL**	
Unprotected sexual activity in adolescence resulting in teenage pregnancies	**Comprehensive adolescent pregnancy prevention programs**
**Inappropriate inter-pregnancy intervals**	Birth spacing and post-abortion contraceptive counseling
Lack of reproductive planning, resulting in unintended pregnancies, unsafe abortions	Contraceptive provision and counseling
Coerced sex and Intimate partner violence	School dating violence prevention programs
Female genital mutilation	Women’s empowerment and community awareness
Substance use (tobacco, alcohol, illicit drugs, excessive caffeine)	
**ENVIRONMENTAL**	
Harmful environmental exposures	

Bold type indicates strong evidence.

## Introduction

Preconception care means providing care before pregnancy is established. Women and couples of reproductive age are generally unaware of the effects that their own health conditions and health-related behaviors may have on the fetus during pregnancy. Although antenatal care is set in the maternal, newborn, and child health (MNCH) continuum [1], it neglects the most critical time of embryonic development, which often occurs before a woman even knows she is pregnant [2]. The evidence increasingly points to earlier care before pregnancy to improve women's health, and better pregnancy outcomes for the mother and newborn [3]–[5].

Preconception care may be defined as “any intervention provided to women and couples of childbearing age, regardless of pregnancy status or desire, before pregnancy, to improve health outcomes for women, newborns and children” [3] or “a set of interventions that aim to identify and modify biomedical, behavioral, and social risks to a woman's health or pregnancy outcome through prevention and management” [4]. For instance, education and awareness about nutritional anemia and congenital malformations can increase receptiveness to and uptake of iron and folic acid supplementation even before pregnancy. The specific aim of preconception care is to improve pregnancy outcomes for mothers and newborns, by optimizing health before a possible pregnancy occurs. Under strict terms, the preconception period may be defined as a minimum of three menstrual cycles prior to the initiation of sexual intercourse, the intent of which is to achieve a wanted and viable pregnancy. An exact “preconception period” has not been standardized by the evidence base; however, since many pregnancies are unplanned, and time to conception for couples varies greatly. We propose that the preconception period be defined as a minimum of one year prior to the initiation of any unprotected sexual intercourse that could possibly result in a pregnancy, reflecting the broader scope of preconception care that extends to adolescents and all women and couples of reproductive age.

A systematic review [3] established that there are currently three levels of evidence within the area of preconception care. For some preconception interventions, such as folic acid supplementation to prevent neural tube defects, the evidence base is strong [6], yet even in developed countries less than half of all women regularly consume folic acid supplements around the time of conception [7]. In other areas, such as intervals between pregnancies, the data shows significant risk in terms of excess maternal deaths, higher rates of prematurity and stillbirths, with short inter-pregnancy intervals 8,9; however, strategies to optimize birth spacing and increase contraceptive uptake are lacking [10]. Finally in women's health, violence against girls and women; unsafe abortions; alcohol and tobacco use; and harmful environmental exposures require further substantiation of magnitude of pre-pregnancy risk, and proof that prevention and management as part of preconception care will have greater impact than prenatal care alone.

Preconception care has the potential to positively impact 208 million pregnancies worldwide each year [11]. Unfortunately, many adolescent girls and women in low- and middle-income countries (LMICs), which have the highest burden of maternal and childhood mortality (map of global infant mortality [World Bank 2011] http://data.worldbank.org/indicator/SP.DYN.IMRT.IN/countries?display=map and map of maternal mortality worldwide [WHO 2010] http://gamapserver.who.int/gho/interactive_charts/mdg5_mm/atlas.html) [12],[13], do not receive the benefits of these interventions, either because they lack access to care or because it is not routinely offered to them before pregnancy. Critical appraisal of the literature review in light of the current global MNCH picture suggests that the greatest benefit would be in these resource-poor countries, and emphasizes the need for implementation strategies and increasing coverage of existing cost-effective preconception interventions.

Although present-day funding for global health is previously unparalleled [14] and a substantial proportion of maternal and child deaths in LMICs are preventable with existing interventions [15]–[17], progress in reducing these deaths is far too slow. Perhaps one contributing factor is the bias that remains in health care and research investment—for example, worldwide 7.6 million children died in 2010, equivalent to global deaths due to cancer and slightly higher than deaths due to heart disease [18],[19], yet funding favors breakthrough research for cancer and heart disease, which have high media interest, while implementation research and delivery for maternal and child health is sidelined. The persisting high mortality for mothers and children in LMICs [20],[21], with its repercussions on global MNCH and overall population health and development, represents a continuing failure and challenge. We assembled a group of maternal and child health professionals whose specific goal was to identify and prioritize evidence-based, equitable research investment opportunities for development and increased delivery of effective preconception interventions in LMIC, with the intent of reducing maternal, fetal, newborn, and childhood mortality and severe morbidity.

## Methods

**Figure 1:**
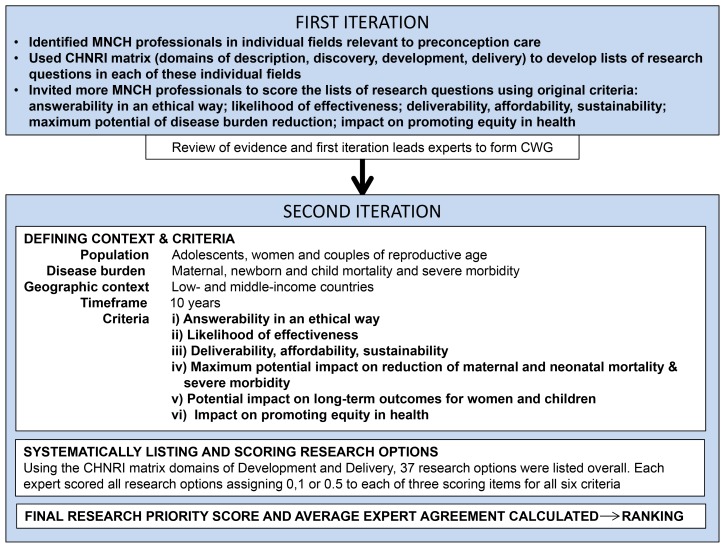
Conceptual framework. Overview of the key steps in this adaptation of the CHNRI methodology.

The Child Health and Nutrition Research Initiative (CHNRI) methodology for research priority-setting was proposed to inform those who develop research policy and/or invest in health research with the aim to impact population health and improve equity in health care [22]–[24]. The process uses a systematic and transparent approach involving an array of health professionals to enlist a wide spectrum of research options relating to a certain health topic and context. Research options are generated in a structured way, using four basic domains: description (epidemiology), discovery (new interventions), development (improving existing interventions), and delivery (health policy and implementation). A priori criteria relevant to the topic are used to score competing research questions in all four domains and then order them in terms of potential influence on health and equity (conceptual framework shown in Figure 1; further details published in previous CHNRI exercises) [25]–[27].

A notable difference from previous CHNRI exercises was the application of the methodology in two iterations. Following an initial consultation coordinated by the Harvard School of Public Health, Aga Khan University, and the Bill & Melinda Gates Foundation, a list of potential experts was drawn up by the investigators in individual fields related to preconception care (such as nutrition, mental health, and infectious diseases) as identified by the initial review. The leads were approached to draw up a list of key questions based on their assessment of research gaps and identify additional subject experts to score the lists of research questions using the original CHNRI criteria: answerability in an ethical way; likelihood of effectiveness; deliverability, affordability, and sustainability; maximum potential impact on burden reduction; and impact on promoting greater equity in health [22]–[24]. Forty-eight of 130 approached completed the scoring (the scored lists may be accessed by contacting the authors), and were geographically representative mainly of Asia, North America, and South America. Many found criterion 4 “maximum potential of disease burden reduction” difficult to estimate, since preconception care to improve maternal and neonatal outcomes is a relatively new avenue. There were also discrepancies in the number and scope of research questions, as well as number of scorers, for each field.

At a meeting of leading international MNCH experts, the potential risk factors and interventions (Box 1) ascertained from the AKU team's systematic review of the literature on preconception care [3 were presented, along with a synopsis of the first iteration. These experts agreed to form a core working group (CWG) to proceed with a second iteration of the CHNRI process for preconception care in a more holistic manner.

**Table:** Box 2. Scoring Criteria Questions

**** ***CRITERION 1:*** ** Likelihood that research would lead to new knowledge (enabling a development/planning of an intervention) in an ethical way.**
1. Would you say the research question is well framed and endpoints are well defined?
2. Based on: (i) the level of existing research capacity in proposed research; and (ii) the size of the gap from current level of knowledge to the proposed endpoints; would you say that a study can be designed to answer the research question and to reach the proposed endpoints of the research?
3. Do you think that a study needed to answer the proposed research question would obtain ethical approval without major concerns?
***CRITERION 2:*** ** Assessment of likelihood that the intervention resulting from proposed research would be effective.**
1. Based on the best existing evidence and knowledge, would the intervention that would be developed/improved through proposed research be efficacious?
2. Based on the best existing evidence and knowledge, would the intervention that would be developed/improved through proposed research be effective?
3. If the answer to either of the previous two questions is positive, would you say that the evidence upon which these opinions are based is of high quality?
***CRITERION 3:*** **Assessment of deliverability, affordability, and sustainability of the intervention resulting from proposed research.**
1. Taking into account the level of difficulty with intervention delivery from the perspective of the intervention itself (e.g., design, standardization, safety), the infrastructure required (e.g., human resources, health facilities, communication, and transport infrastructure), and users of the intervention (e.g., need for change of attitudes or beliefs, supervision, existing demand), would you say that the endpoints of the research would be deliverable within the context of interest?
2. Taking into account the resources available to implement the intervention, would you say that the endpoints of the research would be affordable within the context of interest?
3. Taking into account government capacity and partnership requirements (e.g., governmental intersectoral coordination; partnership with civil society and external donor agencies; favorable political climate to achieve high coverage), would you say that the endpoints of the research would be sustainable within the context of interest?
***CRITERION 4:*** ** Assessment of maximum potential impact on reduction of maternal and neonatal mortality and severe morbidity.**
1. Is this research likely to have substantial impact on maternal mortality and severe morbidity?
2. Is this research likely to have substantial impact on stillbirths reduction?
3. Is this research likely to have substantial impact on neonatal mortality and severe morbidity?
***CRITERION 5:*** ** Assessment of potential impact on long-term outcomes for women and children.**
1. Is this research likely to have substantial impact on long-term health outcomes for women?
2. Is this research likely to have substantial impact on long-term health outcomes for children?
3. Is this research likely to have substantial impact on fulfilling long-term socioeconomic potential of mother and child?
***CRITERION 6:*** ** Assessment of the impact of proposed health research on equity.**
1. Does the present distribution of the disease burden affect mainly the underprivileged in the population?
2. Would you say that either (i) mainly the underprivileged, or (ii) all segments of the society equally, would be the most likely to benefit from the results of the proposed research after its implementation?
3. Would you say that the proposed research has the overall potential to improve equity in disease burden distribution in the long term (e.g., 10 years)?
Questions answered by technical experts to assign intermediate scores for each criterion to competing research options (possible answers: yes, 1; no, 0; informed but undecided answer, 0.5; not sufficiently informed, left blank).

The CHNRI methodology involves four stages (Table S1 provides more information about the modified process, limitations, and validation) [27]:

**(1) Defining Context and Criteria**

An open group discussion was held during the CWG meeting to define the context and criteria, with modifications being incorporated until all CWG members were satisfied. Over 90% of all maternal and child deaths occur in LMICs, particularly South Asia and sub-Saharan Africa [28]. Scaling up preconception care directed at adolescents, women, and couples of reproductive age in such high-burden, resource-poor regions could hasten the decline in global maternal and childhood mortality and severe morbidity through development and delivery of effective interventions. This became the context and target population for the research priority-setting exercise, with a timeline for development and delivery of interventions within 10 years. The CWG emphasized the importance of local or regional MNCH priorities, and underscored that investment in cross-cutting interventions would have greater benefits in improving MNCH outcomes [22],[24].

The criteria were modified to reflect the context and subject of preconception care, with criterion (iv) being amended to “maximum potential impact on reduction of maternal and neonatal mortality and severe morbidity,” and addition of the criterion “potential impact on long-term outcomes for women and children.”

**(2) Expert Input-Listing and Scoring Competing Research Options**

Experts were invited to participate on the basis of their record in maternal and child health research relevant to preconception care. There was an overlap of 11 experts who were involved in both iterations. A serious attempt was made to be inclusive of experts with varied areas of expertise and from different countries (Table S2), with the addition of more experts from Europe and Africa in the second iteration. Experts received a summary of findings with effect sizes from the primary review, along with previously published CHNRI exercises on childhood mortality and stillbirths to help them understand the background literature and priority-setting process. Experts used the CHNRI matrix (Table 1) to develop an extensive list of research options (more expansive research ideas) and research questions (narrower in focus) in preconception care.

**Table 1:** CHNRI matrix.

Research Instrument	Research Avenue	Research Option	Research Question
Epidemiological research: DESCRIPTION	Measuring the burden	Experts were invited to use categorization of research avenues and instruments in Preconception Care to systematically propose a number of “research options”	Experts were invited to propose a number of specific “research questions.” After consolidation and removing of duplicate ideas, 344 questions in the first iteration and 37 in the second iteration were retained for scoring
	Understanding the risk factors		
	Evaluating the existing interventions		
Health policy and systems research: DELIVERY	Studying capacity to reduce exposure to proven health risks		
	Studying capacity to deliver efficacious interventions		
Research to improve existing interventions: DEVELOPMENT	Research to improve deliverability, affordability and sustainability		
Research for development of new interventions: DISCOVERY	Basic research		
	Clinical research		
	Public health research		

Framework from which listing of many research options (level of 3–5-year research program) and research questions (level of individual research papers) were systematically proposed by technical experts.

In the second iteration, the CWG advised that the timelines and criteria for the domains “description” and “discovery” would be inconsistent with those of “development” and “delivery,” and would therefore be ranked inaccurately. A consensus was reached to focus on development and delivery to enable preconception care services to reach women of reproductive age, which would accelerate maternal and child mortality and morbidity reduction in LMICs. Each expert in the CWG presented further information in one area of preconception care most relevant to their own experience and suggested more research options. Short group discussions were held at the end of each session to seek clarification, air dissenting opinions, and outline the most important research options in that area. The chairperson for each half-day session was tasked with drawing out all opinions and promoting clarity, and another member was assigned to document research options and the group's suggestions for emphasis or caveats therein. The lists of research questions from the first iteration were compressed to highlight important gaps, yet still represent the range of research possibilities in preconception care. The final list of research questions was reviewed by the whole CWG at the end of the meeting to ensure that they were framed correctly and comprehensively to allow scoring. The CWG also attempted to ensure that the phrasing of the research questions was not rigid, so for example given the option “What approaches work to increase the use of effective contraception…” research could assess contraceptive counseling to educate and empower women to plan their pregnancies in different settings, or women with and without their husbands, and so forth. Each expert scored the new list, assigning a score to each of 37 research questions in the domains of development and delivery using the six modified criteria (final criteria questions are shown in Box 2, the actual scoring sheet used by experts is shown in Table S3). 24 of 30 scorers participated in the second iteration, which was acceptable as it has been modeled that the CHNRI exercise reaches saturation with 20–25 scorers.

**Table:** Box 3. The Three Highest Scoring Research Questions within Each of the Six Priority-Setting Criteria

**ANSWERABILITY**
What are the public health approaches to regulate and reduce exposures to environmental tobacco smoke? (96)
What is the effect, cost, and feasibility of using cell phones and other information technologies to improve capacities of front-line workers to target and follow up women of reproductive age for improved preconception health care? (95)
What are effective, affordable, and feasible means to screen for diabetes affecting girls and women before conception? (95)
**EFFECTIVENESS**
How can effective interventions to prevent adolescent pregnancy and repeat adolescent pregnancy be delivered at scale? (83)
What are the public health approaches to regulate and reduce exposures to environmental tobacco smoke? (82)
What are the most effective strategies to scale up the prevention/detection/treatment of malaria and helminthiasis to reduce anemia in women of reproductive age? (82)
**DELIVERABILITY**
What are the public health approaches to regulate and reduce exposures to environmental tobacco smoke? (90)
How can cell phones and other information technologies be best utilized to improve care-seeking for preconception services and healthy behaviors, especially amongst adolescents? (87)
What is the effect, cost, and feasibility of using cell phones and other information technologies to improve capacities of front-line workers to target and follow up women of reproductive age for improved preconception health care? (86)
**IMPACT ON REDUCTION OF MATERNAL AND NEWBORN MORTALITY AND SEVERE MORBIDITY**
What effective, affordable strategies could be developed to provide effective STI/HIV identification and management, including early antiretroviral therapy, as part of preconception care, and how could these be adapted to maximize uptake by adolescents? (85)
What should constitute an essential package of preconception health interventions for all girls and women of reproductive age? (83)
How can preconception nutrition interventions, such as diet diversity, micronutrient supplementation/fortification, and achieving optimal BMI, be integrated into broader nutrition and/or health programs and delivered in a cost-effective manner? (82)
**IMPACT ON LONG-TERM OUTCOMES FOR WOMEN AND CHILDREN**
How can preconception nutrition interventions, such as diet diversity, micronutrient supplementation/fortification, and achieving optimal BMI, be integrated into broader nutrition and/or health programs and delivered in a cost-effective manner? (90)
How can a package of promotive and preventive mental health interventions for women and girls be effectively and feasibly provided through community health workers and/or groups, with linkages to the primary health care system for treatment? (89)
What effective, affordable strategies could be developed to provide effective STI/HIV identification and management, including early antiretroviral therapy, as part of preconception care, and how could these be adapted to maximize uptake by adolescents? (88)
**EQUITY**
What effective strategies can be developed to modify individuals' behavior to reduce their environmental exposures to smoke stoves? (95)
What are the public health approaches to regulate and reduce environmental exposures to smoke stoves? (94)
How can the quality of and access to comprehensive post-abortion care services (including contraceptive counseling, provided by different cadres of health care workers, and adaptation to maximize uptake by adolescents) be improved? (89)

CHNRI criteria score: Range from 0 to 100.

The first iteration was conducted entirely via email from March to June 2011. The research options for the second iteration were developed during an expert meeting in July 2011, and the scoring was completed via email by September 2011.

(3) Weighting criteria based on input from societal stakeholders

The scoring criteria may be perceived with varying importance based on the perspective of different stakeholders. For example, parents who experienced a stillbirth may rate mortality reduction higher than a donor organization who may value answerability, or a public health official most concerned with deliverability. For previous CHNRI exercises, a range of stakeholders were polled to weight the criteria [29]; however, the CWG decided not to assign weights for this exercise. Rather, the final rankings are based on the average merit of each research option across all scoring criteria and expert perspectives.

**(4) Computing Research Priority Scores (RPSs) and Average Expert Agreement (AEA).**

Overall RPS was calculated as the mean of scores for the six criteria [23] according to the formula:
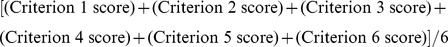
AEA was calculated for each research question as the average proportion of scorers that gave the most common answer while scoring that question:
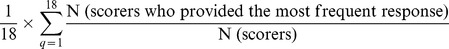
(Where *q* is 1 of 18 criteria questions that experts used to evaluate competing research options).

### Results

Table 2 shows the top research questions (those with RPS>80), of 381 in total (344 questions in the first iteration and 37 questions in the second). Table S4 shows the final scores and ranking of the research questions from the more robust second iteration. The RPS indicates the perceived likelihood that each research option will meet the chosen priority-setting criteria. In the areas of development and delivery of existing interventions, the highest-ranked research option seeks to address the gap in coverage of nutritional interventions, such as supplementation, through integration with other programs. The specified context (expectation of medium term impact in LMIC) allowed research to identify obstacles to delivery of interventions, and research to optimize the use of those interventions, to receive high scores. Priority areas identified were adolescent health, chronic conditions, infectious diseases and immunization, contraception, improving the supply chain for preconception commodities, and public health approaches to reduce exposure to environmental pollutants with adverse MNCH effects.

**Table 2:** The top 12 research questions.

RANK	Proposed Research Question	Answerable?	Effective?	Deliverable?	Mortality/Morbidity Reduction	Long-term Impact	Equitable?	Research Priority Score (>80)	Average Expert Agreement
1	How can preconception nutrition interventions, such as diet diversity, micronutrient supplementation/fortification, and achieving optimal BMI, be integrated into broader nutrition and/or health programs and delivered in a cost-effective manner?	0.86	0.81	0.80	0.82	0.90	0.87	84.2	73.5
2	What are the public health approaches to regulate and reduce exposures to environmental tobacco smoke?	0.96	0.82	0.90	0.76	0.82	0.63	81.7	67.2
3	How can effective interventions to prevent adolescent pregnancy and repeat adolescent pregnancy be delivered at scale?	0.86	0.83	0.76	0.78	0.83	0.83	81.6	70.5
4	What are the public health approaches to regulate and reduce environmental exposures to smoke stoves?	0.92	0.77	0.78	0.71	0.78	0.94	81.6	63.1
5	What approaches work to increase the use of effective contraception, especially long-acting methods, particularly in the postnatal and post-abortion time periods?	0.87	0.78	0.78	0.72	0.86	0.88	81.5	68.2
6	What are effective, affordable, and feasible means to screen for hypertension affecting girls and women before conception?	0.93	0.81	0.83	0.80	0.79	0.69	80.7	68.7
7	What are the most effective strategies to scale up the prevention/detection/treatment of malaria and helminthiasis to reduce anemia in women of reproductive age?	0.92	0.82	0.75	0.70	0.77	0.87	80.7	67.9
8	What effective strategies can be developed to modify individuals' behavior to reduce their environmental exposures to smoke stoves?	0.87	0.75	0.76	0.69	0.81	0.95	80.6	63.4
9	What effective, affordable strategies could be developed to provide effective STI/HIV identification and management, including early antiretroviral therapy, as part of preconception care, and how could these be adapted to maximize uptake by adolescents?	0.81	0.73	0.75	0.85	0.88	0.80	80.4	65.7
10	How can task-shifting to community health workers to screen for chronic conditions among women during the preconception period and take appropriate action (such as referring to specialist, counselling, refer to support groups) be effectively enabled?	0.87	0.76	0.82	0.79	0.80	0.78	80.4	65.2
11	Develop and evaluate the effect and cost of different delivery strategies for an immunization package for girls including rubella and tetanus, and others as appropriate	0.92	0.79	0.83	0.77	0.75	0.77	80.4	67.7
12	How can the supply chain for commodities for effective preconception services (nutrition, contraception, medications for chronic and infectious diseases) be integrated with other logistical systems so that it is more reliable and effective?	0.85	0.74	0.84	0.77	0.83	0.79	80.3	68.2

The highest-ranked research priorities according to their RPS (>80), with AEA.

Three central issues were consistently emphasized, with experts advocating for integration of preconception interventions with other programs and systems; moving beyond the health care setting with task-shifting to community health workers (CHWs); and maximizing uptake of preconception services by adolescents. Addressing these fundamental issues enables interventions to be delivered affordably and sustainably on a wider scale, yet they are rarely considered by research funding agencies.

Although discovery research options were excluded (see “Input from technical experts” above), questions suggested the need to develop simple, cost-effective methods to screen for, diagnose, and treat health conditions that have negative consequences particularly for women of reproductive age. The research questions with the lowest scores reflected interventions for which there is little evidence of effect, such as strategies to promote women's mental health; reduce coerced sex and intimate partner violence; reduce genetic disease risk in the community; and prevent or treat substance use among women and couples of reproductive age. Utilizing information technologies to improve demand for preconception services was also ranked low because of uncertainty about its potential to reduce mortality and severe morbidity.

Discrimination between levels of agreement among scorers on the prioritization of research questions was achieved by calculating AEA (Tables 2 and S4). AEA scores ranged from 60.5% to 84.2%, indicating the proportion of scorers that gave the most common score to an average criteria question for a specific research option. In general, the questions with high AEA were also those that achieved high RPS. Greater points of contention were research options that would require individual behavior modification to improve pre-pregnancy health, or a shift in cultural norms (e.g., economic incentives to increase demand for services, involving men in preconception health, and addressing consanguinity).

The actual scores marked for all research questions by individual experts and calculations for AEA are presented in Table S5. The results exposed how research questions can be prioritized in completely different ways, depending on the criterion used. Box 3 shows the three highest scoring questions for each of the six priority-setting criteria. The most answerable research relates to developing public health approaches to reduce environmental tobacco smoke exposure. This option was also the highest ranked question for deliverability, and scored highly on effectiveness and equity. Other answerable research possibilities were development of diabetes screening tools, and evaluating the feasibility of information technology as an aid for front-line health workers in continuity of care. The ideas most likely to be effective were scaling up interventions to prevent pregnancy in adolescence and anemia in women of reproductive age. The strongest opportunities to improve delivery were assessing the use of cellphones and other information technologies to increase demand for, and promote provision of, preconception care services. The greatest impact on maternal and newborn mortality and morbidity was assigned to developing strategies to identify and manage sexually transmitted infections (STIs)/HIV in the preconception population; deciding upon an essential package of preconception interventions for all girls and women; and studying how best to integrate nutrition interventions into broader initiatives. It was agreed that two of these same research questions would also have the greatest impact on long-term outcomes for women and children. Engaging CHWs in primary preventive and promotive mental health services was another option thought to impact long-term outcomes. Research that would maximally contribute to improving equity was evaluating behavioral and public health strategies to reduce exposure to smoke stoves. It was notably recommended that improved quality and accessibility of post-abortion care services at all tiers of the health system would also improve health equity for women of reproductive age.

### Discussion

This research priority-setting focused on development and delivery of existing interventions during the preconception period in LMICs. The latter has been recognized as a critically important entry point to influence optimal health, nutrition, and birth preparedness in LMICs [30]. The research questions that received the highest scores therefore highlighted the need to develop strategies to increase coverage of basic interventions such as improving nutrition; reproductive planning for adolescents; contraception; prevention, detection and treatment of chronic conditions that affect maternal health; immunization, diagnosis, and treatment of infectious diseases; and reducing harmful environmental smoke exposures. The highest priorities also advocated for a systems-based approach to increase preconception care services in LMICs including integration with other programs; task-shifting to CHWs; improving supply chains for preconception care commodities; partnerships with media and information technology; maximizing demand for and uptake of preconception interventions, especially by adolescents.

The CHNRI methodology aims to ensure that those research options with evidence of true potential impact in the chosen context receive commensurate support from the global health community. The simple, structured scoring method means that those research options that meet most criteria and achieve high expert consensus are ranked highly. Moreover, for each individual research question it exposes strengths and weaknesses through estimations collected from numerous technical experts from various backgrounds. Although the CHNRI process attempts to achieve fairness and greater accuracy in research priority-setting, there are limitations to this method (Table S1). First, the list of research questions developed is not exhaustive and therefore cannot include all possible sound research ideas. Since our primary review focused on health-related interventions in the preconception period, social sector interventions were not emphasized even though these indirectly promote health and wellbeing. In this exercise, education or improving women's literacy was not suggested as a discrete research option although it is an essential means to achieve preconception care and better maternal health. However, this is a major component of other research options that were elaborated, notably adolescent health and pregnancy prevention programs, community-based platforms that target maternal and newborn health in rural areas; and utilizing media and information technology to reach adolescent girls and women with preconception care information and services. Second, the method of expert selection may be seen as the initiator of the process tending to invite other like-minded experts or experts known to them to participate. In this exercise, the initiator invited only the first participants, and then asked them to invite other MNCH experts representing their area of expertise and other geographical regions. In the first iteration, three invited experts were unable to participate in developing research options and 82 were unable to score, with experts mostly commonly citing time constraints as the reason for non-participation. Dissension among experts was reflected in the AEA score, and rearranging research options by this score did not make a significant difference in their priority order since most options with high RPS (>80) also had high AEA (>65). Third, in this application of the CHNRI method, no stakeholder weighting was performed, hence the results are reflective of MNCH experts but do not consider other values that stakeholders might have.

This objective representation of research priorities may be used to guide research policy that is likely to have an impact on the health of women and couples of reproductive age, as well as their young children; and eventually make both research and health care more equitable.

The United Nations Millennium Development Goals (MDGs) aimed to reduce childhood mortality and improve maternal health. While significant progress towards these targets has been achieved, it is recognized that progress in reducing newborn deaths is slow [12] and major challenges remain in reducing maternal mortality [28]. Improving birth preparedness and the health of the mother is a critical step in achieving these targets and has received relatively less attention. The list of research ideas put forth may not seem novel or innovative. Many are already recognized gaps in MNCH. However since research in underlying determinants of health, health policy, and systems, or applied health are rarely appreciated by researchers and investors, this exercise draws new attention to these long-standing concerns. Addressing these issues in LMIC is crucial to build on our success in improving MNCH as we move forward after 2015, the deadline to meet the MDGs

New interventions and strategies, strengthened health systems, quality services, and equity in coverage are needed to confront infectious diseases, chronic conditions, unsafe abortions, and undernutrition, prematurity, and stillbirths, which still threaten maternal and child survival. It is imperative that preconception care is seen as an earlier opportunity, not just for family planning or to reduce maternal and neonatal mortality, but also to improve long-term outcomes for adolescent girls, women, and children. Adolescent health and reproductive health must increasingly be considered as crucial stages in the continuum of care. Health research investment and policy should be pursued in a more balanced way, promoting increased access and delivery of an essential package of preconception interventions. This exercise has led to a concerted global effort led by the World Health Organization to tackle the challenges of reaching girls and women with preconception care services. Reaching a consensus on what constitutes such a package of preconception interventions in LMICs, and investing in implementation research to ensure maximum coverage and uptake should be the next step. Within LMICs, different regions and individual countries may need to further prioritize their MNCH policies and research investment according to their specific causes of maternal and newborn mortality and morbidity and feasibility of scaling up certain interventions.

### Supporting Information

Table S1 **Detailed CHNRI methodology for setting priorities in health research in Preconception Care.**
(DOCX)

Table S2 **Composition of the expert groups.** All participation in this CHNRI exercise was voluntary and carried out without specific funding support. All the experts who were invited to participate had a track record on research in maternal and child health and/or specific fields related to preconception care.
(DOCX)

Table S3 **Template of scoring sheet.**
(XLSX)

Table S4 **The final ranked list of all 37 research questions with scores.**
(XLSX)

Table S5 **The actual scores given by technical experts to each of the research questions for each of the priority setting criteria.**
(XLSX)

## Abbreviations

AEA: average expert agreement
CHNRI: Child Health and Nutrition Research Initiative
CHW: community health worker
CWG: core working group
LMIC: low- and middle-income country
MNCH: maternal, newborn, and child health
RPS: research priority score
