# Supplementary material for: Setting Research Priorities for Preconception Care in Low- and Middle-Income Countries: Aiming to Reduce Maternal and Child Mortality and Morbidity
Source: PLoS Med. 2013 Sep 3;10(9):e1001508. doi: 10.1371/journal.pmed.1001508 (PMC3760783; doi:10.1371/journal.pmed.1001508)
Supplement: Table S1 — Detailed CHNRI methodology for setting priorities in health research in Preconception Care. (DOCX) [file pmed.1001508.s001.docx]

**Table S1:** **Detailed CHNRI methodology for setting priorities in health research in Preconception Care**.

| **STAGE 1: Defining the context and criteria for priority setting**  This application of the CHNRI methodology evaluates the probability of achieving reductions of persisting maternal and child mortality and morbidity through investments in health research. Specifying the context and predetermining the criteria is crucial because disease burden, health priorities and resources may vary substantially in different contexts.  Experts agreed upon the following parameters:  • Burden of interest: Maternal and childhood mortality and severe morbidity.  • Existing situation: Antenatal, neonatal and early childhood healthcare programs and services do exist, yet maternal deaths continue to remain unacceptably high in Sub-Saharan Africa and South Asia. While deaths in childhood have been drastically lowered in the last two decades, neonatal mortality and morbidity is persistently high. Maternal and child morbidity has serious health system, socioeconomic, learning and developmental implications.  • Level of urgency: High (because maternal and neonatal mortality and morbidity continue to remain elevated).  • Population of interest: Adolescent girls, women, and couples of reproductive age during the Preconception timeframe.  • Setting of interest: LMIC where over 90% of maternal and child deaths continue to occur.  • Timeframe: To achieve significant reduction in the rates of maternal and newborn mortality within the next 10 years, as well as a notable decline in other indicators of maternal and child health such as stillbirths and preterm births.  As local circumstances within LMIC also vary, experts were requested to assess research options based on their potential to truly improve maternal and child health on a widespread scale. Therefore, robust ideas were more likely to be highly rated since they would not be as context-dependent. |
| --- |
| **STAGE 2: Choice of technical experts, systematic listing and scoring of research options**  International technical experts with strong expertise and interest in maternal and child health research relevant to preconception care were invited to participate in the process. Experts spanned the continuum of care from women’s health to perinatology, pediatrics, adolescent and reproductive health, and family medicine. Professionals in epidemiology, public health, health policy and programs were represented. Researchers in specific areas relevant to preconception care such as nutrition, environmental health, prevention of congenital malformations, and mother-to-child transmission of HIV were also included. The diverse group of experts allowed for a range of opinions and suggested research questions, helping to minimize bias.  Experts’ then methodically proposed research questions, according to the CHNRI framework (**Table 1**), attempting to ensure that no important research gaps in the area of Preconception care were neglected. The suggested research questions were collated to remove duplications and rephrase language so that the scoring process would be applicable to every research question. Using the initial group of experts as primary contacts to involve other researchers in the field of maternal and child health enabled a representative yield of research questions to be sought from a broad range of expertise.  The second task was to score all research questions independently, according to the priority-setting criteria. For each of the criterion, experts answered three questions with a score of 0, 0.5 or 1 indicating the perceived probability of that research to fulfil the priority-setting criterion (**Box 2, Supplementary Table S5**). |
| **STAGE 3: Community involvement - input from larger group of stakeholders**  The CHNRI methodology involves communities by incorporating the opinions and values from a broader group of stakeholders (e.g. expected recipients of the research, taxpayers who fund health research, health workers, journalists and media, experts in ethics, law, political science, etc.) [26]. Stakeholders lack expertise to directly decide research priorities, but their opinions and values can still be included by weighting the chosen priority-setting criteria according to their perceived importance. For this CHNRI exercise, it was decided that the research questions would be ranked based on their priority scores and average expert agreement. Given the very broad range of stakeholders for Preconception Care, as well as the number of Preconception risks and interventions, the core group of experts agreed that giving each research option an equal chance to be highly prioritised was best. |
| **STAGE 4: Computation of “research priority scores” and “average expert agreement”**  All the experts answered the questions listed in Box 2 with either ‘Yes’ (1 point) or ‘No’ (0 points). They were also allowed to declare an informed but undecided answer (0.5 points) or insufficiently informed to answer the question (left blank). Thus, the proposed research questions got a score for each of the six criteria as "the proportion of maximum possible points scored when an answer was given" (i.e., excluding the missing input), which was a direct measure of collective confidence of the scorers. Each of the listed research questions received six intermediate scores (each ranging between 0-100%), which were then multiplied by 100. The overall research priority score (RPS) was then computed as the mean of all six intermediate priority scores.  The CHNRI process has the capacity to expose which research experts agree should be made a priority, and which research options are more controversial. This promotes more focused discussion among experts, policy makers and other stakeholders following this exercise. The datasets that CHNRI methodology produces are not appropriate for application of the usual Kappa agreement statistics, which has been discussed in detail elsewhere[24]. For each research investment option, the AEA provides information on what proportion of scorers gave the most frequent answer. This parameter accounts for missing answers, is unaffected by responses of ‘undecided’, and would remain unaffected if there was a variation in scorers for the different criteria. In AEA computation, all 4 possible responses (“Yes”, “No”, “Neither” and “Don’t know”) are treated as a valid response. If a sizable proportion of the experts respond with “Don’t know”, the AEA will reflect this as a lower level of overall agreement.  The CHNRI method does not include statistical calculations for power or sample size at the outset of the process. It is based on collective expert opinion and is only semi-quantitative. The RPS and AEA scores are simply indicative of which research questions participating experts gave greater importance to, and whether there was general agreement on the same. For this reason, and to allow flexibility in interpretation of priorities in various local contexts, the RPS and AEA are provided without 95% confidence intervals or another measure of precision.  The exact scores given to all research questions from individual experts are presented in **supplementary Table S3**. |
| Advantages and limitations of the CHNRI methodology  The applied CHNRI methodology has proved to be a successful tool in systematically prioritising a large number of specific research questions, as recently demonstrated in exercises conducted at national level in South Africa, and at global level for mental health research issues, zinc deficiency, childhood pneumonia, childhood diarrhoea, neonatal infections, primary health care, etc. (see <http://www.chnri.org/publications.php>). Benefits of the CHNRI process include its organized approach with clearly defined context and criteria that allows relevant information to be collected from independent expert scorers to discern between research investment options. The CHNRI process outputs are quantitative and are able to be easily understood, so that they are instructive to all those who have a stake in and responsibility to ensuring that health research is relevant and produces real results.  Although the development of the CHNRI methodology was a serious attempt to deal with the inherent complexities of research investment priority setting, some concerns remain about potential biases of the CHNRI approach. In this exercise, the final list included the spectrum of research options relevant to maternal and child health today, yet even though it was systematically produced, it is finite and cannot cover every possible research idea. Specific research methodologies (i.e. randomized clinical trials, etc.) are not clearly outlined in the questions because the research options listed must likely be answered and validated by multiple studies and study types. The CHNRI exercise aims to accurately cover the research spectrum in a given field of study, yet if at the end important questions are deemed to be missing, it is possible to estimate their ranking by comparison to similar questions or scoring by a smaller group of experts.  Another concern regarding the CHNRI process is that theoretically its output may be rather biased since it represents the opinions of a limited group of experts. The number of individuals globally who possess enough experience, expertise and knowledge on Preconception Care and its impact on maternal and child health to evaluate the spectrum of research questions presented is rather limited (although certainly larger than the total group that finally participated). Given that the “sample” of the experts chosen for this exercise was one of the most diverse to conduct a CHNRI exercise to date, while the number of global experts in this relatively new area of Preconception care is not large, it is doubtful that either the list of research questions, or the final scores would change significantly had other experts been polled instead. |
| Validation of the CHNRI methodology  The CHNRI methodology combines two ideas:  (i) “Principal component analysis” - a statistical technique which reduces a very complex system of a large number of variables to a small number of relatively independent “principal components” which still capture a considerable proportion of variation in the system. By defining a set of “criteria”, the CHNRI process converts the intricate and often difficult task of priority setting, which could be approached through an almost infinite number of “lenses”, into an exercise where the key criteria (that are reasonably independent) are underscored and brought into focus.  (ii) “Wisdom of the crowds” – this refers to the process of taking into account the collective opinion of a large group rather than relying on a single individual (or small number of people) to set priorities. It has been demonstrated that the average of a large number of estimates are nearly always closer to the truth than any individual expert judgement. The pre-requisites for this process to function effectively are: (i) Diversity of opinion (each person’s information should be discrete even if it is simply an unusual interpretation of the accepted facts); (ii) Independence (people's own opinions are not swayed by the opinions of others); (iii) Decentralization (people are able to specialize and draw on local knowledge); and (iv) Aggregation (some mechanism exists for turning private judgments into a collective decision – in this case, the CHNRI method).  The validation of the CHNRI method based on the exercises conducted to date showed: (i) extraordinary constancy (correlation coefficients greater than 90%) of scores given to same questions by the same experts at different time points; (ii) nearly identical scores of the same question scored by a larger group multiple times (score always falls within +1.7 points on a scale 0-100); and (iii) Monte Carlo simulations in random sub-samples of the larger group of scorers showed that the probability that the outcomes of the exercise would alter markedly if another group of experts conducted the scoring becomes incredibly small when each criterion is scored by more than 17-23 rational persons with some knowledge of the problem of interest; (iv) varying the context of the exercise leads the same group of experts to assign significantly different scores to the same research questions (Rudan I et al., personal communication).  In this paper, we used 22 technical experts to score each criterion. Thus, given the well-defined context for this CHNRI exercise and a set of simple YES/NO questions, it is wholly improbable that any other group of cogent individuals with some knowledge of maternal and child health, regardless of their background or selection, would ever come to dramatically different conclusions than the present group.  Although this may seem counter-intuitive to some critics, this is the basic property of the phenomenon of collective wisdom, which the CHNRI exercise uses as a central principle. By giving each individual the right and the opportunity to express their personal judgement, and treating the assessment of all participants as equal, the particular biases that each person brings to the process tend to negate and dilute each other, irrespective of participant selection. This leaves only the information based on accumulated knowledge, lifetime experience and common sense of those who took part – which is the result of the CHNRI process.  In other methods where an “expert panel” style approach set priorities, it has often been that one strong voice influences the entire process and outcome, resulting in unequal and inaccurate evaluations that garner support for certain issues at the expense of others that might be more important or urgent. Delphi and CHNRI exercises were conducted in parallel during the large GAPPS meeting (“Global action plan for prematurity and stillbirth”) sponsored by The Gates Foundation in order to make a comparison. The Delphi group leaders realized that since the task of keeping in mind all possible pros and cons of all possible research options was simply too difficult, eventually they would advance the ideas which originally resonated with them and for which they had proceeded to gain support from the rest of the group. In CHNRI groups, however, the systematic nature of the process highlighted the benefits and flaws of many competing ideas, and the top priorities at the end were often surprising to the group because they were novel ideas which therefore were not extensively discussed as none of the group members felt they had expertise in that area. |
