# Supplementary material for: Setting Research Priorities for Preconception Care in Low- and Middle-Income Countries: Aiming to Reduce Maternal and Child Mortality and Morbidity
Source: PLoS Med. 2013 Sep 3;10(9):e1001508. doi: 10.1371/journal.pmed.1001508 (PMC3760783; doi:10.1371/journal.pmed.1001508)
Supplement: Table S2 — Composition of the expert groups. All participation in this CHNRI exercise was voluntary and carried out without specific funding support. All the experts who were invited to participate had a track record on research in maternal and child health and/or specific fields related to preconception care. (DOCX) [file pmed.1001508.s002.docx]

**Table S2. Composition of the expert groups**

| **Name** | **Expertise** | **Country Affiliations*** |
| --- | --- | --- |
| Aimee Webb Girard | Academics (Maternal and infant nutrition in resource-poor settings, nutritional epidemiology) | United States of America, Canada, Nigeria, Kenya, Malawi, Sierra Leone, India, Guatemala, Mexico |
| Ana Langer | Academics, Advocacy (Reproductive health, Maternal health, Perinatal/Neonatal care) | Latin America, United States of America |
| Chandra-Mouli Venkatraman | Advocacy and Policy (Adolescent and Child health) | Switzerland |
| Fernando Althabe | Clinician, Researcher (Obstetrics, Perinatology) | Argentina, Chile, Uruguay, Paraguay, Brazil |
| Igor Rudan | Epidemiology, Genetics, Public health, Researcher (CHNRI priority-setting methods) | United Kingdom |
| Joy Lawn | Researcher, Public Health, Policy and Advocacy (Perinatal/Neonatal care especially stillbirths and prematurity, epidemiology) | South Africa |
| Katherine C Teela | Pollicy (International health, Maternal and Child health) | United States of America |
| Mary-Elizabeth Reeve | Advocacy (Global health, Maternal health, Preconception Care) | United States of America |
| Mireille Toledano | Research, Epidemiology (Environmental epidemiology) | United Kingdom, Western Europe |
| Sohni V Dean | Researcher (Maternal and Child health, Preconception Care) | Pakistan |
| Zulfiqar A Bhutta | Academics, Public Health and Policy (Maternal, Newborn and Child health and Micronutrient deficiencies, Community-based perinatal care) | Pakistan |
| Brian Jack | Clinician, Researcher, Policy (Obstetrics, Family medicine, Preconception care) | United States of America, Lesotho |
| Christopher Howson | Epidemiology, Public health and Policy (International health especially in low-income countries, birth defects and prematurity, Maternal and child health) | United States of America |
| France Donnay | Policy and Advocacy (Maternal health, Reproductive health) | Belgium, Pakistan |
| José M Belizan | Academics (Maternal health, Reproductive health, Obstetrics and Perinatology) | Latin America |
| Josip Car | Clinician, Researcher, Public health (Primary Care, Global health and Health management) | United Kingdom, Slovenia, Croatia |
| Khadija Humayun | Clinician (Pediatrics) | Pakistan |
| Kit Yee Chan | Public Health, Epidemiology, Research (CHNRI priority-setting methods) | Australia, China |
| Majid Ezzati | Researcher (Global environmental health, Exposures to risk factors and outcome on population health and disease burdens) | United Kingdom, United States of America, Switzerland, Canada, Ghana, China, Kenya, The Gambia |
| Reynaldo Martorell | Academics, Policy (Global health, Maternal and Child health, Nutrition in resource-poor settings) | Guatemala, United States of America |
| Stanley Chitekwe | Programmatic implementation (Nutrition and Child health) | Zimbabwe, Eritrea, Malawi, Nigeria |
| Subidita Chatterjee | Public Health (Obstetrics, Reproductive health, Maternal and newborn health, Child and Adolescent health, | India, Thailand, Bangladesh, Bhutan, Nepal, China, South Africa |
| Tanya Doherty | Researcher, Programmatic implementation (PMTCT of HIV) | South Africa |
| Ysbrand Poortman | Policy, Advocacy, Genetics | Netherlands, Europe |
| Zohra S Lassi | Researcher, Epidemiology (Maternal, Newborn and Child health) | Pakistan |

All participation in this CHNRI exercise was voluntary and carried out without specific funding support. All the experts who were invited to participate had a track record on research in maternal and child health and/or specific fields related to preconception care.

*Country affiliations include country of residence and where primary research or clinical experience occurred

Experts who helped develop research questions- First iteration

Aimee Webb Girard, Ana Langer, Atif Rahman, Chandra-Mouli Venkatraman, Chris Howson, Fernando Althabe, Janis Bierman, Juliana Yartey, Kathy Neuzil, Majid Ezzati, Mary-Elizabeth Reeve, Mireille Toledano, Usha Ramakrishnan, Wafaie Fawzi

Experts who completed scoring- First iteration

Chandra-Mouli Venkatraman, Subidita Chatterjee, Kishwar Enam, Natalie Gray, Michelle Hindin, Rajesh Mehta, Sarah Nabukera, Sohinee Bhattacharya, Sorina Grisaru-Granovsky, Manon van Eijsden, Alicia Aleman, José Belizan, Guilherme Cecatti, Guillermo Carroli, Edgardo Abalos, Sergio Hofmeister Martins-Costa, José Ortiz Segarra, Cristina Barreiro, Lorenzo Botto, Christopher Howson, Reynaldo Martorell, Hani Atrash, M.B. Bellad, Eric Steegers, Xu Xiong, Steven Offenbacher, Xu Qian, Azra Ahsan, Alicia Aleman, Claudio Sosa, Muhammad Masood Kadir, Fernando Barros, Sadiqua Jafarey, Sarah Saleem, Mario Aldo Sebastiani, Pierre Buekens, Tatiana Balachova, Lindsay Allen, Mwiru Ramadhani, Yarlini Balarajan, Ibironke Olofin, Ellen Piwoz, Aimee Webb Girard, Xu Xiong
